# Staging the Robot: Performing Techno-Politics of Innovation for Care Robotics in Japan

**DOI:** 10.1080/18752160.2023.2295144

**Published:** 2024-01-18

**Authors:** Giulia De Togni

**Affiliations:** Giulia De Togni The University of Edinburgh, College of Medicine and Veterinary Medicine, Usher Institute, Centre for Biomedicine, Self and Society, Edinburgh, UK email: giulia.de.togni@ed.ac.uk

**Keywords:** Care robotics, techno-politics of innovation, performance, future imaginaries, Japan

## Abstract

In response to the challenges posed by a rapidly aging society and its associated socio-economic difficulties, the Japanese government has encouraged the adoption of AI and robotics technologies for care. Conspicuous investments in these technologies in Japan underscore the dominance of techno-politics of innovation and the advocacy for the robotization of care practices. Such narratives — disseminated by the Japanese state, industry, media, and academia — often overlook the perspectives of the expected users of these technologies. This paper, rooted in a 14-month-long ethnographic study conducted at robotics labs in Japan and the UK in 2022–2023, examines the performance and ethical implications of technoscientific imaginaries portraying Socially Assistive Robots (SARs) as already reliable, safe, and efficient. It sheds light on the intricate relationship between science, technology, the state, and society, emphasizing their use as instruments of power for state-led national development objectives. Moreover, it exposes how technology is presented, creating an illusion of efficiency while neglecting the necessity of involving society in co-designing and co-producing these technologies. The paper ultimately advocates for responsible innovation, emphasizing in particular the need for user involvement to ensure these technologies are not only more efficient and reliable, but also more accessible, inclusive, and fairer.

## 1 Introduction

### 1.1 Performing Techno-Politics of Innovation

“We choose to go to the Moon,” declares a prominent Japanese roboticist while delivering a keynote at a major robotics conference held in Japan in autumn 2022. The roboticist is citing J.F. Kennedy’s Address at Rice University on the Nation’s Space Effort held 60 years earlier, in 1962. The former US president delivered the address to inspire Americans to support NASA’s mission and promised to put an American astronaut on the Moon before the end of the 1960s, which he eventually managed to do with the Apollo 11 Moon landing in 1969. Similarly, on stage, the Japanese roboticist promises that his group of researchers will deliver “AI robots” soon and that these machines will, he argues, “completely transform society.” The roboticist’s animated keynote is followed by the detailed presentations of his team of researchers appointed to create robots enhanced with AI for physical and cognitive support; in other words, the most advanced, reliable, and efficient SARs ever created. More than 3,000 roboticists are attending this conference in person, visiting Japan from all over the world. For many PhD students I meet at the event, this is their first major in-person conference, also due to the COVID-19 pandemic-related travel restrictions that have been in place globally since early 2020. The event has a packed program and on each day of the conference, the atmosphere at the venue is imbued with enthusiasm and positive energy.

On stage, in front of thousands of enthusiastic robotics engineers, the Japanese roboticist delivers a passionate speech imbued with a promising narrative for care robotics. They frantically move across the stage, making ample use of gestures and raising their voice as they call for the “urgent need” to continue investing in “robotics for society.” Recurrent in their speech is the narrative of robots not only supporting but also “transforming” and “saving” aging societies. Their speech flamboyantly resonates in the room and stands out as a performance of technocare promotion, which is particularly representative of the local politics of science and technology for the “public good.” The roboticist uses what has become a common narrative in Japan and which sees robots and AI rescuing the country by 2050, when people aged 65 or older are estimated to reach 36% of the total population (AARP 2022: 2). Such a narrative has been widely used by the Japanese state, media, industry, and academia especially over the past two decades to continue attract funding and actively promote care technologies including IoT (Internet of Things), care robots, and smart devices for private homes.

One hope related to this narrative is that Japanese robotics research will continue also in the future to lead in the development of robotic devices, and that this leadership will help Japan export such technologies to other rapidly aging countries. Such an achievement, several of my Japanese collaborators stressed, may help the country to recover, at least to some extent, from the “three lost decades” (*ushinawareta sanjūnen*) since the collapse of the economy in the early 1990s. Indeed, SARs and other AI and robotic technologies for care are often presented as a solution to shortages of care not only in Japan but also in other rapidly aging, highly industrialized countries such as, for example, South Korea and the UK. Japan is also actively looking at market opportunities in China and India, which have the world’s largest aging populations and as such represent extremely lucrative markets to export robotic technologies for care. As of 2022, Japan was still the world’s number one industrial robot manufacturer, delivering 45% of the global supply (IFR 2022). Moreover, Japan’s export ratio rose to 78% in 2020 alone, when 136,069 industrial robots were shipped and 6% of the Japanese exports of robotics and automation technology were destined for China, which benefited from using robots especially during the COVID-19 pandemic (ibid.).

Since the lucrative robotics industry in Japan represents hope in time of economic stagnation, hyped narratives such as the one produced on stage by this prominent Japanese roboticist—which portrays care technologies like SARs as already reliable, safe, and ready to enter the market—have largely prevailed in the Japanese state, industry, media, and academia. In such narratives, care technologies such as SARs are presented as “the solution” to help “the frail elderly” (Neven 2011), holding on the assumption that robots can compensate for such frailty. Such an approach wrongly assumes that all older adults are frail, when in reality they are not a homogenous group, and they exist in many states of cognitive and physical fitness. To the goal of addressing such supposed universal frailty, however, care is imagined by roboticists as “fragmented” and understood as a collection of tasks that can effectively be programmed into a robot (Vallès-Peris and Domènech 2020). However, to reduce care to a collection of repetitive tasks risks to neglect its complexities and ethical as well as social dimension. Moreover, as I learnt while in the field, we are still far from seeing safe, efficient, and reliable SARs entering the market any time soon. The interviews and observations I carried out at robotics labs in the UK and Japan over 14 months between 2022 and 2023 confirmed this and made me realize the frailty of robots themselves. My collaborators in both countries often remarked that they did not expect to see “intelligent” robots created within their lifespans. At the current stage of the technology, it is the human actors who are “caring” for the robots rather than vice versa. Even in the highly controlled and robot-friendly lab environment, the engineers I collaborated with were constantly worried that they may damage the robot and were always taking extra precautions to prevent the “clumsy” machine from destroying itself or causing any harms to the humans in the room.

Despite the limitations of SARs at their current stage, however, there continue to be conspicuous investments in Japan towards research and development programs in care robotics such as, most recently, the Moonshot R&D Program. The latter was launched in 2020 with an investment of 100 billion Yen (approximately 700 million USD) by the Cabinet Office. The Moonshot R&D Program aims to develop reliable and trustworthy AI-powered robots by 2050 to support aging populations. In addition to large investments such as the Moonshot, since 2020, SARs have attracted attention not only in Japan but also globally as the COVID-19 pandemic created unprecedented circumstances and some noted that these technologies may become key in providing care during a pandemic (Forman et al. 2020). As a result, SARs have increasingly become a topic of discussion in the media and in STS literature. However, already over the past two decades STS literature on SARs had identified wider technoscientific imaginaries of care robotics as either dystopian or promissory.

On the one hand, what have emerged in STS literature over the years are narratives of a dehumanized, “cold” and emotionless robotic care (Sparrow and Sparrow 2006), incompatible with and rival to human care (Hülsken-Giesler 2017; Turkle 2011); on the other hand, what have also come to light in the STS literature are promissory discourses which recognize that SARs may become valuable aids for caregivers by raising efficiency and safety levels (Treusch 2015) and mediating “warm” qualities of care work (Breazeal 2002; Pols and Moser 2009; Pols 2012). Most recently, some STS scholars have also argued that SARs have the potential to “care” for humans through social interaction, physical assistance, and therapy delivery (Chita-Tegmark and Scheutz 2020). However, this body of literature has focused mainly on imaginaries and cultural influences on expectations towards and responses to SARs, while often neglecting what the technology entails for care values and practices.

This paper focuses on the Japanese context and stresses how science and technology continue to be used primarily as a form of power and as political instruments to serve the state-led national development, staging the technology to create an illusion of efficiency, and neglecting what this entails for care values and practices. The underlying assumption is that end-users will have to adapt to the finished product, as neoliberal governmentality dictates. The idea of “governmentality” was first introduced by Michel Foucault in 1991, when the French philosopher drew attention to the processes by which the conduct of a population is governed by institutions including the state. Drawing on Foucault’s analysis, several scholars (including e.g. Barry et al. 1996; Burchell 1996; O’Malley 1998; Rose 1996) have focused on how governments create mechanisms that work “all by themselves” to bring about governmental results through the devolution of risks onto the individual (the so-called “responsibilization” of subjects). As such, neoliberalism produces a political rationality that determines the ways in which governments manage people’s actions through “technologies of domination.” This paper calls for the need to criticize such technologies of domination, and to co-produce AI and robotic technologies for care together with end-users.

In terms of structure, the paper is divided into three sections. First, the introduction provides background information on the challenges raised by an aging society and the role of technologies such as IoT, robotics, and AI to tackle such challenges. This section also contextualizes the research carried out by the author, addresses key arguments in STS, and outlines this study’s conceptual framework. Then, the methodology section that follows focuses on the fieldwork’s methods, including collection, selection and analysis of the data. Finally, the third section offers critical analysis of ethnographic data from interviews and observations conducted at robotics labs in Japan, connecting these findings to the key arguments outlined earlier. Results and discussion are combined in this section, which concludes by stressing the importance of including end-users in designing and coproducing care technologies.

### 1.2 Staging the Robot

In his book “Administering Affect: Pop-Culture Japan and the Politics of Anxiety,” anthropologist White (2022) explores how “pop-culture diplomacy,” soft power ideologies, and nation branding strategies—otherwise known as “Cool Japan”—have emerged in the twenty-first century and continue to influence policy in Japan to this day. Pop-culture started influencing policy in Japan in the early 2000s and has continued to play an important role in nation branding throughout the 2010s and early 2020s, White argues. He explains this in terms of pop-culture representing “a hopeful vision for Japan’s cultural resurgence after nearly three decades of economic stagnation and geopolitical anxiety” (ibid.: 1). The underlining idea in the book is that pop-culture can transform widespread anxiety into a hopeful vision for Japan’s cultural resurgence in a time of geopolitical anxiety due to shifts of power in East Asia. As such, pop-culture becomes political and reflects the worries of administrators who have become hypersensitive to perceptions of Japan’s declining political prestige in the world.

In a similar way to how pop culture has been promoted and instrumentalized for economic and political reasons, over the past two decades, public discourses around the future potential and imagined impacts of AI and robotics for care also have reached extreme heights of aspiration for fundamental transformations of Japanese society into a high-tech utopia. In the narratives produced by the Japanese state, industry, media and academia, robots represent new hope for the country’s rapidly aging population, low birth rate, and longstanding economic stagnation. While in other aging countries, such as the UK, relationships with robots are generally considered to be less intimate and accepting, in Japan bonding with inanimate objects and cohabiting with a “friendly robot” appear to be deeply embedded in the local culture (Hornyak 2006; Kim and Kim 2012; Tamura et al. 2004). This, however, is the product of decades of technical discourses and practices of robotics researchers carefully adapting their design to public taste to promote social acceptance of their work (Frumer 2018; Šabanović 2014). The hyped discourses produced by the Japanese state, industry, media, and academia have also greatly contributed to these hyped narratives.

Notably, the advocates of care robotics in Japan are often the same as those who support pop-culture, namely politicians, policy makers, administrators, entrepreneurs, and scientists who are disproportionately older and male. Whereas pop-culture’s representative images are often those of young, *kawaii* (“cute”) women. In the case of SARs, these machines are also often given a female, reassuring voice. Such design choices reflect particular gendered visions that remain prominent in Japanese society. This phenomenon draws attention on the underrepresentation of women in research and development in the country. Notably, only 13.8 per cent of the members that created the “Social Principles of Human-Centric AI” in Japan were women (Asia Pacific 2020); and women make up only 16.7% of Japan’s research workforce (academic staff at national universities) compared with the OECD average of 40% (Hori 2020). While women remain underrepresented, the voices of male actors continue to promote certain views and sentiments over others for Japan’s pop-culture and AI/robotics innovation.

Furthermore, images of friendly robots, such as the popular anime characters Doraemon and Astro Boy, proliferate in Japanese pop-culture and the media, influencing people’s views and opinions on the technology. Many of the Japanese roboticists I collaborated with mentioned how they have been influenced by these anime characters, which they loved as children and still appreciate as adults. Some of them stated that their *yume* (“dream”) was, in fact, “to recreate Doraemon and Astro Boy, make them real.” Such anime characters are still widely used in Japan to promote robotics in the media. This is how soft power operates, having “the ability to affect others by attraction and persuasion rather than just coercion and payment” (Nye 2017: 2). Soft-power discourses can turn cultural production into political capital; culture itself, as a result, becomes a resource to exercise power. This use of “culture-as-resource” (Yúdice 2004: 1) is certainly not unique to Japan. For a comparison in East Asia, for example, South Korea has also recently captured international attention through the proliferation of high-tech products as well as its pop-culture commodities of K-pop, TV dramas, and films.

This celebration of pop-culture and tech industry in both Japan and South Korea has been instrumental in attracting the interests and investments of local governments and international companies, as well as to persuade the public that technologies such as SARs are safe and reliable. As Frumer and Šabanović explain in their commentary to this special issue, the promotion of SARs in both South Korea and Japan is part of the complex politics of *mise-en-scène* technologies. In film production, *mise-en-scène* (“setting the stage”) refers to the meticulous stage design and arrangement of actors, setting, props, costumes, and lighting. Frumer and Šabanović draw particular attention on how robots are “staged” in Japan and South Korea as part of a performance, a show, a spectacle, an illusion that portray the technology as functional and efficient. Drawing on such illusion, soft power discourses of technocrats continue to present SARs as a panacea in both countries. However, what are the implications of the politics of *mise-en-scène* technologies?

In their analysis, Frumer and Šabanović refer to two cognitive mechanisms, namely “resonance” and “detachment” from reality. The “illusion” that robots may rescue aging populations works well in both Japan and South Korea because the design of robots has been adapted in both countries over three decades to resonate with the local end-users’ taste and culture. For this illusion to work, however, as Frumer and Šabanović point out, some degree of detachment from reality becomes necessary. Although technologies like SARs are far from being reliable and efficient, policy makers and ethics panelists continue to promote them. This is a political choice to justify, legitimize and continue to attract investments in research and development while refusing alternatives to these technologies, such as for example increasing the number of immigrant care workers (Wright 2019). Science and technology are future-oriented endeavors, often entangled with the promise of benefiting society and improving the quality of human life. However, these promises are instrumental and political as they become part of a narrative of what is timely, urgent, and desirable and hence worth investing in. To ensure that such hyped narrative produced by the state, industry and the media is believable, scientists “need to stage what is not yet possible or certain by performing a vision as if it was already real” (Lipp 2022: 4). The work of scientists in the lab hence becomes political and an essential part of the performance needed to stage the robot.

Since the 1980s, STS literature has used the metaphor of theatre staging to illustrate how scientists carefully prepare and present their work. Notably, this literature has borrowed terminology from Goffman (1956), who uses the metaphor of the theatre to explain social interaction, the presentation of the self in everyday life, and the difference between front stage, backstage, and off-stage behaviors. For example, Latour (1988: 86–87) referred to the “theaters of proof” used by Pasteur in front of different audiences to persuade people that his method was scientifically valid. Then Alač et al. (2011) explained how engineers stage the robot to become “social” by actively directing the attention of experiment participants towards the machine. Moreover, Treusch (2015: 203) has shown how robotics engineers often explain failure during robotic experiments in front of external audiences in terms of fallibility traits “legible as human.” In other words, they anthropomorphize the robot to make it appear “human-like” and justify the machine’s faults.

Furthermore, Möllers (2016) has used the expression “techno-scientific dramas” to refer to the ways in which scientists carefully stage their work to attract funding. Bischof (2017) also explains how engineers shield their robots from complications that they cannot solve by carefully preparing the *mise en scène* of their experiments. And, most recently, Lipp (2022) has shown how “robot dramas” are carefully enacted as engineers carefully stage testing environments to create an illusion of efficiency. This paper focuses on the performance of techno-politics of innovation for care robotics in Japan, looking at how the technology is staged, who is left out in the process, and what this entails for the future of care practices. In the section that follows, I outline in detail the methodology adopted for this study. The methodology section is then followed by the results of the research, which are integrated with the discussion.

## 2 Methodology

This paper is based on 14 months of ethnographic fieldwork (qualitative interviews and observation sessions) which I carried out at robotics labs in Japan and the UK between April 2022 and June 2023. I interviewed 60 robotics engineers who were developing and prototyping SARs. Of these, 30 participants were based at Japanese institutions (leading universities and prestigious research centers collaborating with local governments and the industry); whereas the other 30 participants were based in the UK, although they very often had experience of collaborating with Japanese partners (academia and/or the industry) and of carrying out research projects in Japan. Moreover, while in the field, I also carried out 20 interviews with end-users and spent 40 weeks doing observation sessions at robotics labs and assisted living facilities in both countries.

In Japan, I was hosted as a visiting researcher at a robotics lab at The University of Tokyo and I also had the pleasure to work closely with key collaborators based at other major research institutions including Waseda University, Kyoto University, Osaka University, Tohoku University, and AIST (National Institute of Advanced Industrial Science and Technology). While carrying out this research, I was invited to contribute to a couple of projects, which were part of the Moonshot R&D Program. This unique opportunity offered me valuable insights into the Program and its projects as well as access to its teams of researchers, who warmly welcomed me in their labs and greatly informed my project. I am extremely grateful to all of them for their generous support and the valuable expertise they have shared with me. To protect their identity, the names of my collaborators have been omitted in this paper.

The 80 interviews that I carried out for this study were one-to-one conversations carried out in English or in Japanese, depending on the interviewees’ preferences. All interviews were audio-recorded after receiving consent from the participants. Each interview lasted between 60 to 90 minutes and was held in person or online to accommodate my collaborators. After introducing my project and answering any questions they may have, I asked all interviewees to tell me about their background, research projects, engagement with, and interest in SARs. I then asked about their views on the current state of the technology, including its limitations, what they think is cutting hedge, and what still needs to be improved. Moreover, I asked them what they think are the drivers for the development of these technologies and their perceptions of how SARs may impact on care practices. Finally, I asked about their hopes and concerns for the future of care robotics; and whether they themselves would be open to using a care robot to make any aspect of their life easier.

To account for their experience of being interviewed by a social scientist and discussing about the ethical and societal impact of their work and/or experience interacting with care robots, I concluded each interview by querying whether the discussion played out as they imagined it might, and if they had anything they would like to add. Many respondents, especially experts in the field of robotics, admitted they had never thought about these issues prior to being interviewed and some added they were surprised to realize the impact their work may have on care practices and society at large. Overall, I received very positive feedback from the interviewees, with several follow-up emails from them inviting me to meet again and continue our conversations, and think about potential future collaborations.

Following to the interviews’ collection and transcription, I conducted analysis iteratively combining my field notes and interview transcripts and drawing on a range of approaches to data analysis, including but not limited to critical discourse analysis (CDA), linguistic anthropology, and thematic analysis. Although this ethnographic study led to the collection of a wealth of data, for the purpose of this paper I selected a limited amount of case studies. Data selection was done based on thematic analysis, selecting the data that referred more closely to the topic of this paper, namely the performance of techno-politics of innovation in Japan and the staging of the robots. Hence, I focus here on the narratives produced by the engineers working on the robots. During the study, I did not come across anything that would contradict the findings I outlined in this paper. However, as this study is based on a limited number of participants—most of which are male engineers with abled bodies, based at prestigious research institutions—it presents some limitations.

In addition to the interviews and observation sessions I carried out at robotics labs, I regularly met with a small group of key participants, all robotics engineers (10 in the UK and 10 in Japan), to discuss informally about their work. These were all early career researchers, in their late 20s and mid 30s, who were carrying out experiments with robots at the labs I was visiting. The fact that I was about their age, fluent in both English and Japanese, and an early career researcher myself, greatly helped facilitate our interactions. Moreover, my status as an STS researcher working on AI and robotics innovation allowed me to be perceived as an active participant of the interaction in the lab rather than just an external observer. In particular, it helped facilitate “ethnographic conversations” (informal interviews) with my key collaborators including discussions about responsible innovation and ethics, but also their work-life balance (or lack thereof) and the power dynamics in place at their lab. I was regularly invited to join them for lunch on campus or at nearby restaurants as well as to take part in extra lab activities for team building, including team sports. I welcomed these opportunities and have greatly benefited from spending time with these talented, highly motivated, and ambitious researchers. I have learnt a great deal from all of them and I would like to express here my sincere gratitude to them for this.

In terms of the technologies considered for this study, SARs that my collaborators in the UK and Japan were implementing included, for example: humanoid robots for triage in hospitals and for use in care facilities; pet-looking robots used for companionship, and to detect if, for example, the user falls and automatically call an ambulance; exoskeletons used for rehabilitation to help people with physical impairments and care workers who need to lift care recipients. When referring to SARs, I include in such definition exoskeletons and lifting robots following the suggestion of Wright (2018: 36), who points out that not only care robots but also exoskeletons and lifting devices “have social effects and impact on the socially constructed meanings and practices of care.” Indeed, drawing on my experience in the field, I agree that all these robotic devices have the potential to transform care practices and care relationships.

In STS literature, Turkle (2007) has described SARs as “relational artifacts,” sociable machines equipped with computational systems designed to create a conduit for “emotional touch” with humans by actively facilitating smooth communication. Other STS scholars also posit that “socially embodied robots” (Ziemke 2001) have the potential to fulfil users’ psychological and emotional needs, including interaction, communication, companionship, care for others, and emotional attachment (Kolling et al. 2016). These “caring machines” may have a wide range of applications including providing social, emotional, and cognitive as well as physical rehabilitation, encouraging healthier lifestyles, reminding people to take their medications, delivering tele-medicine support, and providing companionship to residents of care homes to reduce feelings of loneliness (Kidd and Breazeal 2007; Lara et al. 2017; Pineau et al. 2003; Robinson et al. 2014; Wada and Shibata 2007). However, although there is much potential for these devices, in this paper I argue that it is crucial to also understand the current limitations of SARs as well as the ethical and social implications of the hyped techno-politics of innovation that keep promoting these technologies.

## 3 Results and Discussion

### 3.1 Let the Drama Begin

While in the field, I observed engineers working relentlessly at robotics labs to carefully adjust the scene for the robot to perform the tasks (almost) exactly as planned. In Japan, for example, I regularly visited a research lab in a leading university where a group of robotics engineers were programming a bulky humanoid robot to perform activities such as folding clothes and cooking. Supposedly the robot would be later deployed in private homes, under the slogan: “One smart robot per person, accompanying them for a lifetime” (*hitori ni ichidai isshō yorisō sumāto robotto*). During one of my visits, the researchers kindly offered me the opportunity to remotely control the right arm of the robot by using bracelets with sensors on my right arm. The robot would have then moved its mechanical arm following my exact movements.

Before starting, the researchers carefully showed me how I should move my arm “to avoid damaging the robot.” They explained to me that I should make movements “extremely carefully” and “slowly” to prevent the robot from hitting surrounding objects and damaging its fragile mechanical body. They also warned me multiple times that I should not approach the robot and stay at least two meters away from it, for my own safety. They themselves moved to a separate room during the experiment, observing me and the robot from a window. As I started performing the task, extremely carefully and slowly as instructed, I saw the researchers from the other side of the window holding their breath. After a few seconds, the robot slowly started following my movements and the researchers appeared relieved and pleased. One of them told me: “Great, keep moving, yes … Slowly, carefully.” To break the tension, I decided to wave at them, of course still slowly and carefully, using my robot avatar. Surprised by the sight of the robot waving “hello” to them, the researchers burst into laughter. As one of them removed the bracelets with sensors from my right arm at the end of the experiment, they said to me: “That was the first time someone who is not from our lab used the robot, thank you for using it with care. It can be very dangerous, you know, it is big and has a lot of strength, and yet it can also break very easily. It is clumsy and a bit unpredictable sometimes, so we do get stressed when we do these tests.” As this example shows, and as my collaborators often stressed during their interviews, SARs are not ready to be safely deployed yet in an environment that is not completely supervised and carefully adjusted for them.

Robots are tested under highly controlled conditions inside the labs and in assisted living facilities that are made robot-friendly. In these venues, obstacles such as carpets are removed, sensors and cameras that help the robot orientate itself are installed everywhere, and the light is carefully adjusted and optimized to ensure the robots’ visual sensors can work. During my regular visits at robotics labs, I was not only looking at the machines but also focusing on the human actors who were carefully preparing the *mise en scène* to create an illusion of efficiency for the robots. Robotics labs are environments aimed at optimizing robots’ performance to appeal to the industry and lay audiences during public demos. However, what happens in a lab is extremely difficult to replicate in an uncontrolled and unpredictable environment such as e.g. a private home, care facility, or hospital. Moreover, as the great majority of robots do not have any AI components yet, it is humans—namely the software engineers who wrote and fed algorithms into the machine—who perform the key actions during the experiments and not the robots. If this is not disclosed during the tests, and the user (lay person invited to the lab to try using the technology) is unaware of this form of control, the illusion engineers create results in particular techno-scientific imaginaries of robot’s capability, suggesting that the machine is more capable that it actually is. A common criticism to this approach is that Wizard of Oz experiments focus not on human-robot interaction but rather on human-human interaction *mediated* via a robot. Addressing these issues, some scientists are calling for more transparency and reality checks.

One of my collaborators, a robotics engineer with decades of experience in the field of robotics and in charge of a large group of researchers in the UK, was invited to give a talk at a major university in Japan in winter 2022. At the talk, as they were showcasing the several research projects ongoing at their lab in front of the Japanese academic audience, my UK collaborator openly addressed the limitations and vulnerabilities of robotics and questioned whether robots will ever become fully autonomous. They asked the audience: “The real challenge is, can we wait until they become fully autonomous if they will, ever?” My collaborator then drew attention on the need for “shared autonomy,” where robots and humans collaborate. The latter acknowledges the fragility of robots and the need for engineers and end-users’ intervention. In the context of care, Lammer et al. (2014) describe this cooperation between care recipients and SARs as “mutual care.” My collaborator concluded their talk by stressing the need to reconsider hyped narratives and start working instead on “more realistic” projects that involve shared autonomy:

“Scientists often stress what goes well and do not address what goes wrong. I believe we need to be more open and honest about our research and stop hiding what does not work. No one will trust you if you hype your research data. What we need is to understand where the system fails to make any progress.”

During their presentation, my UK collaborator drew attention not only on the technology per se but also on the societal challenges that robotics as a field may help to address. These include sustaining care workforce in rapidly aging societies; decommissioning operations in toxic and dangerous environments such as the Fukushima Daiichi nuclear power plant, where a triple meltdown took place in 2011; rescuing victims in disaster zones; and helping build a more flexible re-deployment after the COVID-19 pandemic through e.g. sustaining remote working. The audience in the room, mainly male Japanese professors, silently listened to the talk and only at the end one of them asked:

“Everything you said is interesting, of course. But I run a lab and I am extremely busy, you know, like you. My question is, how can we keep the momentum? I mean, the hype, the interest in these technologies.”

The discussion that followed addressed the pressure to attract funding and the considerable investment of labor that engineers (globally) have to do in order to showcase their robots as reliable and efficient, and as such still worth the investments. Having to prioritize “keeping the momentum,” there is not much time left to think about societal impact and the ethical dimension of their work, the person from the audience seemed to suggest. To which, my collaborator from the UK replied admitting that their work as the head of a robotics lab has indeed involved over the years “many sleepless nights.” My collaborator and their research team in the UK are busy throughout the whole year, and especially when there is a major conference or an industry event as they need to prepare paper presentations and demos to attract funding, build new collaborations, and keep high the reputation of their lab. Indeed, amidst all these commitments, finding the time to think about the societal impact and ethical dimension of their work can be challenging; but this is still, nonetheless, necessary, the UK roboticist concluded. The Japanese roboticists at the talk nodded in silence, yet the questions that followed focused merely on technical aspects such as machine learning and computer vision to enhance robots’ reliability. No one else further problematized how technoscientific expectations and hyped narratives are navigated and managed by roboticists. What emerged from this verbal exchange between the two roboticists is that to keep “the momentum,” heads of robotics labs in both the UK and in Japan feel the pressure to continue performing certain narratives that may help them secure the existence (and success) of their labs.

During my fieldwork in both countries, my collaborators often referred to how prominent roboticists act in public “like politicians” to promote the technology through using hyped promissory narratives, suggesting that robots are more capable that they actually are. In the words of one of my Japanese collaborators who was describing their head of the lab and supervisor in Japan: “During my PhD I seldom saw him. He did not really mentor me. But, you know, he acts like a politician [*seijika*] and actively promotes the work at the lab. He does that very well, securing funding for all of us and this is very important, I think. So, I am grateful for what he does.” This was a recurrent narrative for many of my key collaborators especially in Japan, where the early career researchers I closely collaborated with seldom saw their line managers and heads of their labs. The latter, according to my collaborators, were “too busy” attracting funding, speaking to the media, and dealing with the industry and governmental agencies. There were some exceptions, however. Amongst the heads of the labs I met in Japan, one in particular mentioned that they recognized how important it was for engineers to “stop and think” about how their work may impact society. They told me:

While reading journals and attending conferences, I always come across these positive narratives towards care robots written by policy makers and other researchers; but what about those who will use these technologies? What is really needed by someone affected by ASD [autism spectrum disorder], for example? And what is needed, conversely, by someone affected by dementia or other cognitive impairments? Even care givers often do not really know what the answers to these questions are. What needs does the person really have? Only the person themselves will know this, right? However, the design of these technologies is being done without even knowing the end-users’ side. The common approach here in Japan, but also elsewhere, as far as I know, is to develop the technology first and then to ask the user to adapt to it. It should be the other way around; that is why the end-users’ input is so important. Are robots matching people’s real needs? We need more qualitative research involving end-users to account for their subjective needs and for the feelings they may have towards these technologies.

Techno-politics of innovation and the narratives they produce are imbued with biased views of how the future should look like, reflecting dominant institutional power. Whereas, as this Japanese roboticist pointed out, the voices of lay people, in this specific case end-users of SARs, often remain unheard. Adding to the complexity, care practices (the ways care is provided and received, and the meanings attached to it) as well as the ways aging and neurodiversity are perceived and conceptualized are culturally specific. For example, expectations towards elderly people and those affected by ASD can greatly differ across cultures. As a result, the levels of independence expected for these populations and the kind of technologies being developed to sustain their living can also greatly differ. In relation to people affected by ASD, for example, this Japanese roboticist stated:

Here in Japan, we receive an education that makes us all look the same. Individual differences in other countries such as the UK or the US are perceived as positive traits since they make someone an individual; but here in Japan differences are not perceived as good characteristics. So, people with ASD are likely to face more struggles here in Japan. However, research has shown that people affected by ASD often feel more at ease for example when communicating online rather than in person. Changing the environment conditions can help them to open-up and communicate better. The kind of technologies we aim to develop at our lab reflect this approach; we start from the needs of end-users rather than imposing on them societal views of how someone should act. However, I am not aware of anyone else adopting this approach in Japan.

Even though over the seven months of my visit I did not observe any participatory design workshops or similar activities involving end-users at this lab, my collaborator stated they intended to take into account end-users’ views in their research. Their frustration with the lack of end-users’ involvement in Japanese academia resonates with dominant technoscientific imaginaries which expect society to adapt to technology rather than the other way around. Many of my collaborators—especially in Japan—remarked that activities involving end-users were rare, if not inexistent. When I presented my research at their labs and called for the need to codesign and coproduce these technologies with end-users, roboticists in both countries seemed genuinely interested and asked me how they could do that in practice. However, they also often remarked how difficult it was for them to engage more with lay audiences since they were already overwhelmed due to their ongoing research projects. In particular, in the case of early career researchers, my collaborators in both countries were expected to publish peer-reviewed papers and deliver presentations by strict deadlines while many of them were on fixed-term contracts and barely managing to “survive in academia.” Indeed, during my fieldwork, many of the young engineers I collaborated with eventually left academia and moved to the industry, where they could get open-ended contracts and much higher salaries.

The early career researchers I met, whether PhDs or postdocs, barely had enough time to complete the tasks they were given by their line managers and supervisors. In particular in Japan, where work-life balance is poorer than in the UK, my collaborators were not only spending Monday to Friday at the lab from early morning until late evening, coding non-stop to ensure that the robot would not fail when the important day of the demo comes; it was also not rare for them to stay at the lab overnight, during weekends, and even national holidays. On top of their hectic research schedules, early career researchers often had teaching and caring responsibilities. Some of my collaborators in Japan also had side part-time jobs to pay the bills, as their scholarships (if they were lucky enough to have funding) were not enough to make ends meet. Many of them were still living with their parents, as they could not afford paying the rent for an apartment, especially in Tokyo. Notably, this was not the case for the established academics mentioned earlier in this paper, as they already had obtained tenure and had gained a position of leadership, managing their own lab at prestigious universities and research institutions.

My collaborators in the UK, who had a relatively better work-life balance compared to colleagues in Japan, often told me that they would like to do more to include end-users in their projects. However, many complained that they already struggled to find the time to complete mandatory tasks for their research, and that it was unrealistic for them to find the time to organize non-mandatory activities such as participatory design workshops, especially considering the cumbersome ethics process they would have to go through. In the UK, the NHS (National Health Service) requires researchers to spend months of work to gain ethical approval to carry out a study involving human subjects. This was particularly discouraging for PhD students who wanted to complete their PhD in time. In addition to the above, my collaborators in both countries mentioned that they were also concerned about safety issues and how to ensure that lay people invited to their labs would not get hurt when carrying out experiments with robots that are heavy, clumsy, and still highly unreliable.

In fact, over 14 months of fieldwork, I struggled to find any participatory design workshops or a similar activity at the labs I visited in both countries. In Japan, these were almost inexistent and in the UK they were rare. Perhaps this was, at least in part, due to the fact that I carried out fieldwork soon after the restrictions of the COVID-19 pandemic were lifted. While in the UK activities were “back to normal” when I started fieldwork in April 2022, in Japan everyone was still wearing masks and social distancing was still a requirement when I was visiting for my research between October 2022 and April 2023. During the seven months I spent in Japan for my fieldwork, I asked all my collaborators about any participatory design workshops or similar activities happening at their labs. The researchers often appeared puzzled when I asked them this question. Many said they were not aware of “anything like that” happening at their institution and a few of them asked me to explain what a participatory design workshop is. To my surprise, I came to realize how unusual or even unknown these activities are in Japan despite the hyped promotion of care technologies in the country. As one of my Japanese collaborators later confirmed to me, bursting into laughter when I asked them this question, “you will not find anything like that here in Japan.”

When I asked my Japanese collaborators why they would not try to engage with the public, in addition to their hectic schedule, many often reported the difficulties they assumed they would encounter when interacting with end-users due to the gap in lay-expert knowledge communication. They also remarked that they did not have any incentives from their supervisors, line managers, or the heads of their labs to engage in such outreach activities which would require considerable time and effort and, at the end of the day, were not mandatory. In the UK, the importance of carrying out activities involving end-users is now widely recognized and funding agencies are increasingly encouraging applicants to include public engagement activities in their projects. The labs I was collaborating with in the UK were indeed making some efforts to increase their public engagement and outreach activities accordingly. However, the focus at the robotics labs I visited in Japan still entirely remained on delivering the product fast to meet pressing deadlines rather than involving end-users in early design and development. If this does not change, however, robots will continue to be produced in the lab far away from society, without any input from those they are supposedly created to help in the first place.

### 3.2 Science and Technology as a Form of Power

Scientists based in Japan and involved in ambitious innovation research are expected to deliver soon care robots that are reliable, safe, and trustworthy. However, most of the robotics engineers I interviewed admitted they did not expect to see such robots created within their lifespans. Nonetheless, the Japanese government, industry, media, and academia continue to stress for the need to promote economic growth and industrial competitiveness through the rapid development of care technologies such as SARs. Similarly, as outlined in other contributions collected in this special issue, also in South Korea official visions of science and technology have increasingly “become interwoven with enduring projects of nation building” (Kim 2015: 154). Drawing on disputes over three different case studies (namely nuclear power, the regulation of biotechnology, and the import of US beef during the Bovine Spongiform Encephalopathy outcry), Kim (ibid.: 153) highlights how, while activists’ groups have tried to resist official visions of development, in prevailing “sociotechnical imaginaries” in South Korea science and technology are still seen primarily “as a form of power” and “as instruments to serve state-led national development.”

Dominant techno-scientific imaginaries determine how risk, technology and health should be managed. Citizens who disagree with such imaginaries and try to resist them often face strong criticism from the authorities as well as fellow citizens, as unpatriotic and unwilling to suppress their concerns and fears for the “public good.” This was the case for anti-nuclear activists in South Korea (ibid.) as well as for anti-nuclear activists and nuclear evacuees from Fukushima, whose concerns were silenced in public discourse after the triple disaster (earthquake, tsunami, and nuclear crisis) of March 2011 (De Togni 2021). As Kim (2015: 166) points out, dominant sociotechnical imaginaries define “the risks and benefits of science and technology in society predominantly in terms of implications for the future prosperity and empowerment of the nation.” Immersed in such imaginaries, political elites and the scientific community believe that higher degree of environmental, health and safety risks shall be tolerated and left to tackle at a later stage, prioritizing the rapid national development through the utilization of science and technology. This, however, leaves the citizens to deal with the risks, as neoliberal governmentality dictates.

This paper has highlighted the relationship between power and techno--scientific imaginaries produced in relation to SARs in Japan. The study calls for the need of coproducing these technologies together with end-users to ensure that they become not only safer, more efficient, and trustworthy but also more accessible, inclusive, and fairer. To analyze how SARs are being conceived and developed in Japan and what kind of issues this research and development approach raises, the paper has engaged with and problematized local hyped narratives and performances of techno-politics of innovation. Although a variety of often contrasting imaginaries may coexist within a society, it often falls to governments, policy makers and the media, as well as other institutions of power such as academia, to select and elevate certain imagined futures above others. Inevitably this situation creates specific visions of futures, which can be more desirable for certain groups rather than others. In the case of SARs, as well as other AI and robotics technologies for care, while the views of the Japanese government, industry, media, and academia appear predominantly optimistic and widely supportive of these technologies, what is often ignored are the views of the people who are supposed to use these devices. To what extent are these technologies accessible and inclusive? Will they become affordable in the future, or will only the wealthiest part of the population benefit from using them? Or, perhaps, will only the wealthiest among us have access to human care whereas the rest of us will be left with care robots? Will robots enhance or replace human care? Will they simplify the work of caregivers or make it even more cumbersome? How will they transform care practices and the ways in which human care is valued and perceived? More qualitative work is urgently needed to answer these timely questions before care technologies are implemented in society and it becomes too late to influence innovation processes.
